# Association of electroretinography with visual outcomes after ophthalmic artery chemosurgery for retinoblastoma in ICRb D and E eyes

**DOI:** 10.1371/journal.pone.0210647

**Published:** 2019-01-16

**Authors:** Ariana M. Levin, Jasmine H. Francis, Molly McFadden, Scott Brodie, Y. Pierre Gobin, David H. Abramson

**Affiliations:** 1 Memorial Sloan Kettering Cancer Center, Ophthalmic Oncology Service, New York, United States of America; 2 University of Utah, Moran Eye Center, Salt Lake City, United States of America; 3 Weill Cornell Medical Center, New York, United States of America; 4 NYU School of Medicine, Department of Ophthalmology, New York, United States of America; Massachusetts Eye & Ear Infirmary, Harvard Medical School, UNITED STATES

## Abstract

**Importance:**

Predictions of visual outcomes are useful in clinical and family decisions regarding treatment for retinoblastoma. Very little has been published on the association of post-treatment visual acuity with pre-treatment electroretinography (ERG), which can be performed on infants too young to reliably quantify visual acuity.

**Objective:**

To report associations of pre-treatment ERG with post-treatment visual acuity in eyes with advanced retinoblastoma treated with ophthalmic artery chemosurgery (OAC).

**Design:**

Retrospective case-control study of eyes treated from 2006 through 2017, with mean follow-up of 51 months (range 2.3–150 months).

**Setting:**

Single large academic center.

**Participants:**

Group D and E eyes treated with OAC at Memorial Sloan Kettering Cancer Center with recorded visual acuity and ERG (30Hz flicker).

**Main outcome and measure:**

Snellen visual acuity (uncorrected) compared to initial 30Hz flicker ERG.

**Results:**

This study included 157 Group D and E eyes. Results of the Jonckheere-Terpstra test for trend were statistically significant and indicated that eyes with lower pre-treatment ERG readings tended to have more visual impairment post-treatment. Among eyes with initial ERG 75+ μV, 11 of 32 eyes (34%) had visual acuity 20/40 or better. Among eyes with ERG 0 μV, 44 of 46 (96%) had visual acuity of 20/200 or worse.

**Conclusions and relevance:**

Eyes with advanced intraocular retinoblastoma treated with OAC can achieve excellent visual acuity, but poor ERG at initial visit is associated with poor visual acuity after treatment in the majority of eyes. Expectations regarding visual potential may influence decisions about treatment.

## Introduction

Expectations regarding visual potential can influence physicians and families making decisions about treatment regimens for retinoblastoma, including enucleation versus treatments that potentially save the eye. Very few studies of ocular success using primary multi-agent systemic chemotherapy include reports of final visual acuity, and even fewer have reported initial or final electroretinography (ERG). In particular, there is a paucity of studies on visual outcomes in International Classification of Retinoblastoma (ICRb) Group D and E eyes. The majority of D and E eyes worldwide are primarily enucleated, because disease is difficult to cure with systemic chemotherapy. For D eyes salvaged with systemic chemotherapy, visual acuity is usually poor. Several studies report that only one tenth of such eyes attain 20/40 or better (Table 1). [1–8] In the past, clinicians felt that the potential risks of treatments were not justified by what they predicted would be poor visual outcomes. [9] However, ophthalmic artery chemosurgery (OAC; often combined with intravitreal chemotherapy) has dramatically changed the outcome for both D and E eyes over the past decade. [10]

**Table 1:** Published series reporting Snellen visual acuity in eyes with retinoblastoma. Included series were published 2006 or later (concurrent with our study population), listed in order of cohort size (RE = Reese-Ellsworth classification).

**Authors**	**Publication**	**Primary treatment**	**Number of eyes**	**Classification**	**Enucleation**	**% 20/40 or greater**	**% 20/50 to 150**	**% Poor vision**	**Fix/Follow**	**Follow-up (months)**
Levin et al	(present)	OAC	157	ICRb D or E	39 (25%)	19 (12%)	5 (3%)	133 (85%, 20/200 or worse)	NA	51
Levin et al	(present)	OAC	63	Unilateral ICRb D or E with macular disease	11 (17%)	4 (6.3%)	1 (1.5%)	58 (92%)	NA	
Levin et al	(present)	OAC	18	Unilateral ICRb D or E with no macular disease	4 (22%)	5 (28%)	1 (5.6%)	12 (67%)	NA	
Berry et al	*Pediatr Blood Cancer*. *2013 Apr;60(4)*:*688–93*.	IVC plus EBR	62	ICRb D	17 (27%), included	5 (8.1%)	6 (8.3%)	31 (50%, 20/200 or worse)	20 (32%)	54.2
Berry et al	*Br J Ophthalmol*. *2014 Aug;98(8)*:*1061–5*.	IVC plus EBR	52	ICRb D	Enucleation excluded	5 (9.6%)	12 (23%)	25 (71%; worse than 20/200)	10 (19%)	50
Shields et al	*Ophthalmology*. *2009 Mar;116(3)*:*544-551*.*e1*	IVC	38	ICRb E	Enucleation excluded	4 eyes (11%, 20/50 or better)	11 (29%, 20/60 to 20/100)	21 (55%, 20/200 or worse)	2 (5.3%)	63
Choi et al	*J Korean Med Sci*. *2010 Apr;25(4)*:*546–51*.	EBR	32	RE II-V	8 (25%), included	9 (28%)	5 (16%)	18 (56%, 20/200 or worse)	NA	150
Fabian et al	*Am J Ophthalmol*. *2017 Jul;179*:*137–144*.	IVC plus adjuvent	32	ICRb D	Enucleation excluded	7 (22%)	9 (28%)	16 (50%, worse than 20/200)^a^	NA	64.4
Rao et al	*Br J Ophthalmol*. *2018 Apr;102(4)*:*490–495*.	Intravit. injection	17	ICRb C and D	1 (5.8%), included	6 (35%)	5 (29%)	5 (29%, 20/200 or worse)	NA	23.8
Schefler et al	*Ophthalmology*. *2007 Jan;114(1)*:*162–9*.	IVC and laser	14	RE I-V	Enucleation excluded	5 (36%)	3 (21%)	6 (43%, 20/200 or worse)	NA	36
**Authors**	**Publication**	**Primary treatment**	**Number of eyes**	**Classification**	**Enucleation**	**% 20/40 or greater**	**% 20/50 to 150**	**% Poor vision**	**Fix/Follow**	**Follow-up (months)**
Kim et al	*Korean J Ophthalmol*. *2010 Dec;24(6)*:*347–52*.	IVC plus local	13	ICRb B	Enucleation excluded	5 (38%)	2 (15%)	6 (46%, 20/200 or worse)	NA	79.3
Tsimpida	*Br J Ophthalmol*. *2013 Nov;97(11)*:*1464–70*.	IAM	12	(excluded eyes with foveolar disease)	0 (0%)	6 (50%)	1 (8.3%)	4 (33%, 20/200 or worse)	1 (8.3%)	21
Jung et al	*J Pediatr Hematol Oncol*. *2018 Apr 20*.	Proton beam rad.	4	ICRb D	2 (50%), included	1 (25%)	0 (0%)	3 (75%, 20/200 or worse)	NA	12; 50

^a^ This number was calculated by the authors of the present study from the numbers reported in the published study.

In previous attempts to standardize assessment of visual function, we used ERGs taken before and after OAC as a proxy for visual function. However, we recognize that ERGs do not substitute for visual acuity assessment. Visual acuity after treatment with OAC has so far only been reported in small numbers. Fabian *et al* reported on five Group D eyes. [11] Tsimpida *et al* reported on twelve eyes, excluding eyes with disease at the foveola. [12] The purpose of this study was to report associations of ERG with long-term visual acuity after treatment with OAC for Group D and E eyes.

## Methods

Eyes with retinoblastoma treated with OAC at Memorial Sloan Kettering Cancer Center from May 2006 through February 2017 were identified. Inclusion criteria were age at least 36 months at last follow-up, visual acuity and ERG (30Hz flicker) data available in the electronic health record, and at least three months follow-up after treatment completion. Memorial Sloan Kettering Cancer Center Institutional Review Board approval was obtained. The IRB waived the requirement for informed consent for this retrospective review. 30 Hz flicker ERG recordings were obtained under anesthesia. Data were collected retrospectively.

Primary outcome was Snellen visual acuity (uncorrected) measured after completion of an OAC treatment course (defined as a period that included OAC and adjunctive treatments without a treatment-free period lasting 3 months or more) in eyes grouped by ERG recorded at initial visit. Secondary outcome was Snellen visual acuity grouped by ERG in eyes with macular disease (tumor or detachment) versus eyes without macular disease. Reported visual acuity was not best corrected acuity, because refraction was not always done at the time of examination.

Ordered groups of ERG and Visual Acuity were created: ERG was grouped by levels (0, 0.1 to 24.9, 25 to 49.9, 50 and up), and VA was grouped by levels (20/100 or better, 20/150 to 20/800, CF or HM, LP only, NLP or enucleation). These groupings were chosen as such (rather than a strictly clinical cut-off, e.g. driving vision or legal blindness) to balance the number of eyes per group. The Jonckheere-Terpstra (JT) test, a nonparametric trend test appropriate for ordinal groups, was used to evaluate the effect of ERG upon VA. The sign of the Z statistic of the normal approximation of JT statistic was used to determine the direction of the effect. Due to sparse data and some 0 cell counts, an exact version of the JT test was used where the p-value was calculated using a Monte Carlo estimate. Figures were designed to reflect clinical cut-offs, with enucleated eyes categorized as no light perception.

## Results

This study included 157 Group D and E eyes of 149 patients (81 with unilateral disease; 68 with bilateral disease). Median age was 20 months at first clinic visit (range: 0.4–103 months; mean: 24 months) and 6.2 years at visual acuity measurement (range: 3.4–14 years, in salvaged eyes; mean: 6.7 years). Mean follow-up was 51 months (range 2.3–150 months). Eyes received median of 3 OAC infusions (range: 1–9; mean: 3.6). Median maximum dose of melphalan, topotecan, and carboplatin were 4 mg, 0.5 mg, and 30 mg, respectively. Median cumulative dose of melphalan, topotecan, and carboplatin were 12 mg, 1.6 mg, and 80 mg, respectively.

Among patients with unilateral disease, the results of the JT test for trend were statistically significant p = < .0001 and the sign of the Z statistic was negative, indicating that those with lower ERG readings tended to have more visual impairment. (JT = 749.0, Z = -4.1, p = < .0001, N = 81). Among patients with bilateral disease, the results of the JT test for trend were statistically significant p = 0.0003 and the sign of the Z statistic was negative indicating that those with lower ERG readings tended to have more visual impairment (JT = 700.5, Z = -3.5 p = 0.0003, N = 76).

**Fig 1:**
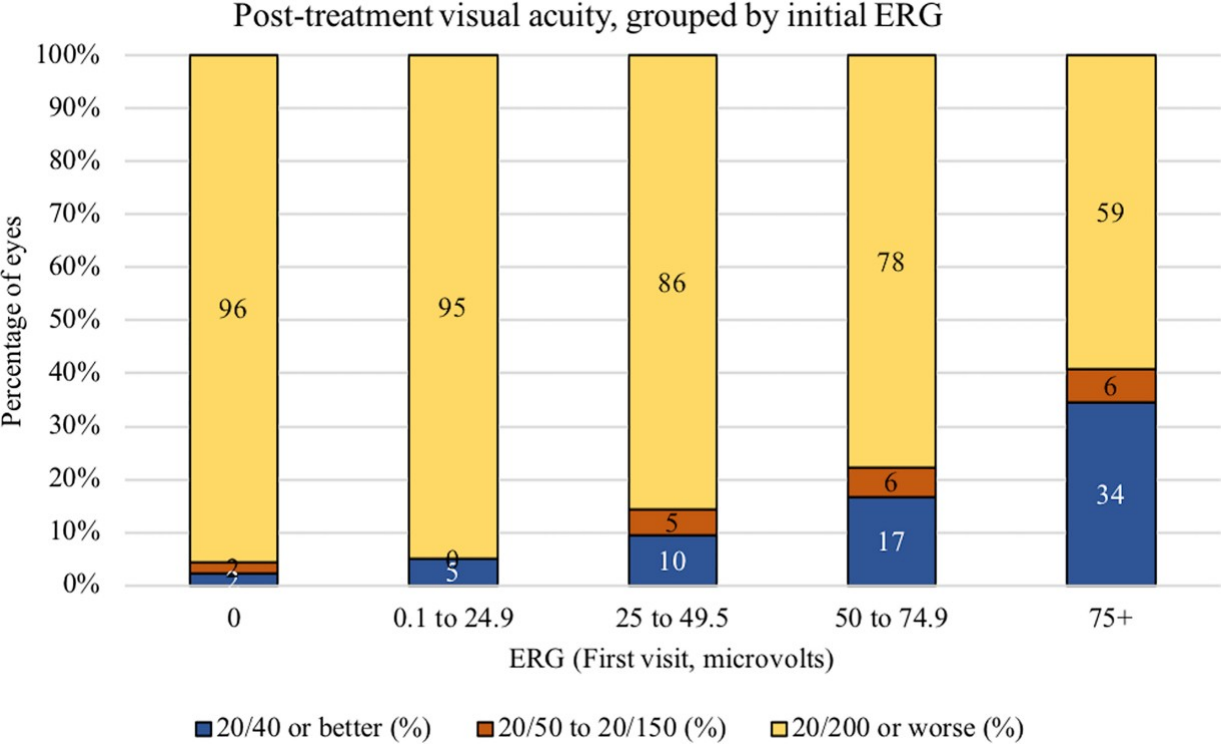
Visual acuity after ophthalmic artery chemosurgery (OAC) for retinoblastoma, grouped by ERG at initial clinic visit.

**Fig 2:**
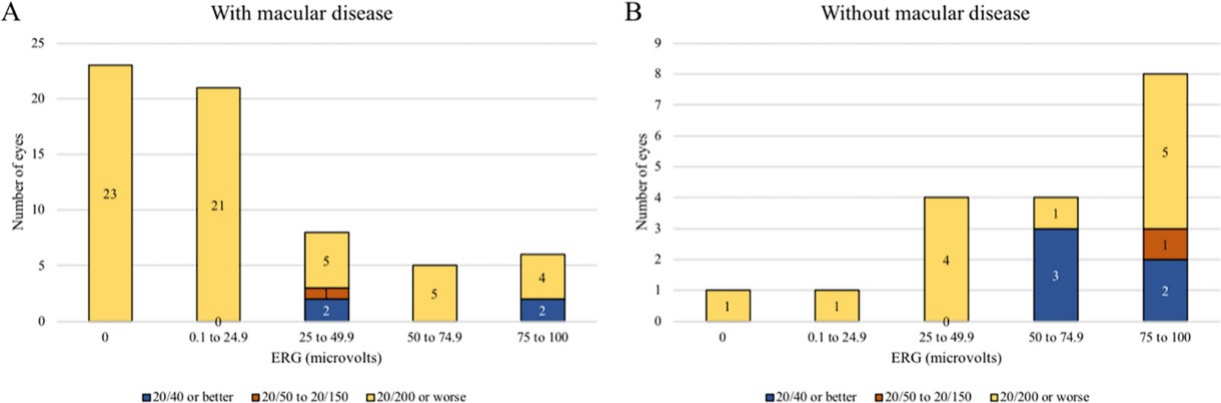
**Visual acuity grouped by initial ERG in eyes with A) macular disease or B) no macular disease.** Only patients with unilateral disease are included. There were not enough patients with bilateral disease and no macular disease for a meaningful comparison.

Visual acuities grouped by initial ERG and presence of macular disease are shown in Figs 1 and 2. Eleven of 32 (34%) eyes with initial ERG measuring 75 μV or higher achieved visual acuity of 20/40 or better, contrasted with 1 of 46 (2%) of eyes with initial ERG of 0 μV and 2 of 40 (5%) eyes with ERG 0.1–24.9 μV.

## Discussion

This is the first report associating pre-treatment 30Hz flicker ERG with final visual acuity in eyes with advanced intraocular retinoblastoma treated with OAC. Eyes with advanced disease (D and E) traditionally have been enucleated because of the difficulty in saving such eyes and the feeling that the risks of treatment do not outweigh the benefits, but following treatment with OAC most of these eyes can now be salvaged and many of these eyes have excellent vision. Among Group D and E eyes without macular disease, 5 of 18 eyes (28%) achieved 20/40 visual acuity. If the macula has tumor or detachment at diagnosis, overall visual acuities are worse than those without macular disease. Thus, initial ERG measurements and macula status are prognostic of vision for most eyes. However, some eyes predicted to have dismal outcomes may end up with useful vision.

Although most eyes with poor ERG ultimately had poor visual acuity, 3 of 86 eyes (3.5%) with ERG less than 25 μV achieved 20/40 visual acuity or better. Thus, eyes with advanced disease and poor ERG measurements prior to treatment with OAC do have potential for useful vision following treatment. This result supports a conclusion from our previous study: retinal function can recover in eyes with minimal baseline ERG. [13]

It is impossible to compare our results to prior reported series of eyes treated with systemic chemotherapy, because almost 90% of our patients with D and E eyes are treated in our center without enucleation. [14] In most centers, the majority of such eyes are primarily enucleated, inducing a selection bias. Some prior series even exclude Group E eyes because they are so advanced. Lastly, measurements of *best-corrected* visual acuity may yield different results and make comparisons between studies difficult.

In conclusion, our study demonstrates that eyes with advanced intraocular disease treated with OAC can achieve excellent vision, but poor ERG at initial visit is associated with poor visual acuity after treatment. Information regarding visual potential can be helpful in counseling patients and families regarding treatment decisions.

## Supporting information

S1 Table ERG, visual acuity, macular involvement and laterality of disease. (XLSX)
